# Affordable and Sustainable Biosensing Technology

**DOI:** 10.7150/ntno.106495

**Published:** 2025-02-27

**Authors:** Pranjal Chandra

**Affiliations:** Laboratory of Bio-Physio Sensors and Nanobioengineering, School of Biochemical Engineering, Indian Institute of Technology (BHU) Varanasi, Uttar Pradesh - 221005, India.

## Abstract

A growing need for rapid disease detection worldwide highlights the importance of innovative approaches to ensure prompt diagnosis and more effective patient care. As of now, various nanobioengineered systems have been developed and applied for disease diagnosis at an early stage, and a few are in clinical trials currently. However, the affordability and sustainability of diagnostic systems “Biosensors” are still being questioned in the translational research. To overcome these impediments and accelerate translational research, my laboratory is trying to develop sustainable, clinically relevant, nanobioengineered platforms for the detection of disease biomarkers at minimum cost.

## Transition from paper-based optical sensors to electrochemical sensors and their translational aspects

Paper-based sensors epitomize a cutting-edge detection technology, offering sensitivity, versatility, affordability, flexibility, and sustainability^1^-^3^. Our goal was to make an alkaline phosphatase (ALP) monitoring kit out of paper that could easily and accurately detect pasteurization as well as diagnose several diseases. The developed paper-based optical sensor and detection through it was a big step toward making diagnostics easier to get and cheaper, and this study was published in a very reputed journal^4^. This experience also laid the foundation for our work in electrochemical sensing, where we have made advanced devices used for many things, such as detecting infectious diseases and keeping an eye on biomarkers for long-term conditions^5^-^8^. Later, our group shifted their attention toward developing an electrochemical sensor for detecting ALP in milk with improved sensitivity and response time^9^. Our lab always aims to use electrochemical sensors to get high sensitivity, specificity, and quick reaction times, all of which are important for early diagnosis. Going from paper-based optical sensing devices to electrochemical sensing devices has made us even more determined to connect complicated lab tests with real-life clinical uses, making technology easier for more people to access and use in healthcare^10^-^12^. Nowadays, our research work mainly focuses on making devices that are small, portable, and inexpensive by combining nanomaterials, enzymes, and other novel components^13^,^14^. My research group devised a portable ALP detection system that can be easily deployed in any dairy or beverage industries and requires only 15 seconds to acquire data^15^. Recently we have been granted a patent (Patent No: 521579) for this important contribution, and it is now on the verge of commercialization. Developing safe and reliable diagnostic devices has become an essential need for disease diagnosis with minimum cost investment. My ultimate aim is to contribute to India's translational research and to set new goals for the advancement of affordable technologies. Many of our developed technologies are well recognized around the globe, and our lab is making every effort to deliver safe, reliable, and cost-effective technologies for everyone around the world. We have always been interested in using biochemical methods to find and treat illnesses and in bridging the gap between lab research and clinical use. In particular, my ongoing research is in this direction only, as we are developing affordable and easy-to-get biosensors and nanoelectronic devices that will reach even an underserved group. Working with doctors, government agencies, and business professionals to make high-tech diagnostic tools popular in homes is one way we want to make sure that our innovations improve people's lives and meet the needs of healthcare in the future.

## Role of the mediator in fostering translational research and key challenges of translational research

There has always been a gap between the viewpoints of lab researchers and medical professionals^16^, ^17^. We need to find someone who can bridge the gap between research laboratories and medical hospitals because many times medical doctors do not know real research problems and several times researchers do not know real medical problems, though both have broad ideas to solve major healthcare problems. Managing these two different roles by a single person is difficult; however, somehow, we have to bridge this gap. There comes the mediator, whose role in translational research is to connect what scientists find in the lab with what doctors need to know in order to find real healthcare answers. At its core, translational research is about making sure that scientific discoveries help real people and communities, and this is where mediators put their attention. As part of our job, we try to make diagnostic tools that are not only based on strong science but also useful, cheap, and able to be used in clinical settings. We want to make it easier for budding researchers to bring an effect on the real world by working with clinicians, business partners, and regulatory bodies. One of our main goals is to bring cutting-edge and affordable biosensors and diagnostic devices from the lab to clinics, especially for the ones that do not have a lot of resources. For good translational research, we need to know about real-world scientific problems, which are prior needs in healthcare. Thus, this is how I visualize my role in translational research, where I am acting as a mediator in bridging the gap between lab and clinic for improving patient health.

Translational study is very important for improving healthcare, but it has to deal with a lot of problems^18^. The gap between scientific discovery and clinical application is one of the main problems that we have covered in detail above. This gap is often caused by researchers, clinicians, and business stakeholders not being able to communicate and work together well. Each group has its own goals and schedules, which can make it harder to develop and use new technologies on time. The regulatory landscape is another very important problem. It can be hard for small study teams or new businesses to figure out how to follow the complicated rules that govern medical devices and diagnostics. Making sure that rules are followed while still being innovative often takes a lot of money and time, which can stop potential solutions from reaching the market. Another problem that keeps coming up in translational studies is funds. Projects that bridge the gap between the lab and the clinic can have a hard time getting funding, especially in the beginning stages when results are still scarce. Lack of funds can stop people from coming up with new ideas and slow down the process of making and testing new technologies. Lastly, it is important for successful translational practices to consider the needs of a wide range of groups. Solutions must be easy to use and reasonably priced, especially in areas with limited resources. Making sure that new treatments and tests are fair and can reach the people who need them the most is hard and needs careful teamwork and community involvement. We can improve translational methods and, in the end, patient outcomes by recognizing and dealing with these problems.

## Impact of publications in knowledge sharing and innovation and the essence of delivering affordable biosensors for doorstep diagnostics

There are several reasons why publications are important in scientific study and translational medicine. They make a formal record of our results so that the broader community stays updated and can evaluate, replicate, and build knowledge upon our work 19. This transparency also makes it easier for researchers to understand the research trends, influence collaboration, and speed up innovation, all of which are important for broader societal impact, for example, improving healthcare solutions in our case. Publications also share information with healthcare professionals and policymakers, making sure that important new developments reach the people who can use them. A strong publication record also boosts your professional reputation and makes it easier to get funding and work with others. Finally, publications help set the direction of future research by showing where we still need to learn more. This is how healthcare innovation happens. They are basically the backbone of science because they make it easier for people to share knowledge and make real progress in translational research.

Of course, it is essential to come up with a way to send cheap biosensors straight to every corner of India, especially in villages, for real-time and doorstep diagnostics^20^. The important thing is to make diagnostic tools that are cheap, simple, and do not need a lot of training or equipment. We are heading in this direction in order to achieve the aforementioned goals. Working together with local health care workers and using mobile health tools can make training and distribution more effective. Making deals with non-governmental organizations (NGOs) and government programs can also help get these biosensors to the people who need them the most. Making diagnostics easier to get will give people in rural areas the power to keep an eye on their health and get medical help when they need it, which will eventually improve the quality of healthcare.

## Conclusion

The development of sensing technologies for rapid disease diagnosis is progressing, with sustainable and reasonably priced sensor devices emerging as a pivotal advancement. In this editorial, various aspects of translational research along with the importance of affordable and sustainable biosensing technology have been described. More focus has been highlighted on nanoengineering platforms as point-of-care diagnostic systems. Although there are several limitations to transferring technology from the laboratory to the patient's bedside, affordability and sustainability are the most significant ones. Nevertheless, we have outlined in this editorial several innovations in which we constructed an affordable and sustainable sensing technology for biomarker detection and are on the verge of translation. Further, the role of mediators in the regulations of the translational bridge has been briefly discussed. In the end, the importance of publications in advancing novel ideas as well as the affordable biosensors for the revolutionization of diagnostics has been discussed, which opens new directions in science and technology. While the advancements in this field so far show an optimistic trajectory, this editorial serves as a call for the scientific community to concentrate more on developing sustainable and reasonably priced biosensor platforms, working with healthcare professionals to narrow the gap, and commercializing them to transform healthcare.

## Author's inspiration and biodata

Prof. R. C. Srivastava, a Humboldt Fellow, has inspired author Prof. Pranjal Chandra for his scientific journey. Prof. Srivastava has taught him a lot about values and how to be dedicated to study. His toughness and dedication to learning have been driving principles for Prof. Pranjal all through school and college life. Also, Prof. Kamal, who taught Prof. Pranjal the importance of science, was a big reason why Prof. Pranjal chose to become a teacher and do research. He made Prof. Pranjal love academic discipline and education. Prof. Pranjal's PhD professor, Prof. Yoon-Bo Shim, was a game-changer in his life. He introduced Prof. Pranjal to the world of sensors, which is now an important part of his work. His supervision and help made Prof. Pranjal realize how powerful devices could be in improving health care. Along with this, Prof. Pranjal also gets motivation from his students; they fuel his desire to connect science with real-world applications, with the goal of making important contributions through translational research.

Author, Prof. Pranjal Chandra is currently Head and an Associate Professor, School of Biochemical Engineering, and Faculty In-Charge of a virtual Sustainable Development Centre at the Indian Institute of Technology (BHU) Varanasi, India. He earned his Ph.D. from Pusan National University, South Korea, and did post-doctoral training at Technion-Israel Institute of Technology, Israel. With over 12 years of professional experience, he focuses on interdisciplinary research in biotechnology, nanobiosensors, and biochemical diagnostics. He is supervisor / co-supervisor of 14 PhD Students (5 Awarded and 9 Ongoing), 2 DST National Post Doctoral Fellow (NPDF), 8 M.Tech / MS (Awarded), and several undergraduate / training students. His group has designed several commercially viable biosensing prototypes that can be operated for onsite analysis for diverse applications. He has two international patents (South Korea), one national patent, and has authored over 194 journal articles/ invited chapters in top-tier journals/books. He has published 20 books on various aspects of biosensors /diagnostics/material Engineering from IET London; Springer Nature; CRC press USA and many books are in press/under preparation. Chandra's work has been greatly highlighted / featured in over 450 news agencies/media outlets globally (India, USA, London, Canada, Dublin, China, Malaysia, Singapore, China, Taiwan, etc.,) including Chemistry World News Cambridge, London; Rajya Sabha TV; DD Science; NDTV; News Nation; The Hindu; Times of India; Science Trends USA; Nature India; Vigyan Prasar, DST Gov of India; Global Medical Discovery, Canada; Cover News in APBN Singapore; Business Wire, Dublin, etc. Work done by Chandra's team has also listed /covered / featured in the 12 Innovative Sensors Globally of all kinds in 2019 by: BUSINESS WIRE, London & Ireland; Associated Press, New York City, USA; Frost and Sullivan, Texas, United States, etc. His work on "Smartphone Integrated Milk Testing Paper Sensor" has also received appreciation from the Honorable HRD MINISTER, Government of India, Dr. Ramesh Pokhriyal Nishank Ji. Dr. Chandra is recipient of many prestigious awards, coveted honours, and fellowships such as; Fellow of Indian Chemical Society (FICS), Prof. B.K. Bachhawat Memorial Young Scientist Lecture Award 2022 by the National Academy of Sciences (NASI), Prayagraj, India, Shakuntala Amir Chand Prize 2020 by the Indian Council of Medical Research (ICMR), Eat Right Research Award 2022 by the Food Safety and Standards Authority of India (FSSAI), DST Ramanujan fellowship (Government of India); Early Carrier Research Award (ECRA) (DST, Government of India); BK -21 and NRF fellowship, South Korea; Technion Post-Doctoral Fellowship, Israel; Nano Molecular Society India Young Scientist Award; Biotech Research Society India (BRSI) Young Scientist Award; Young Engineers Award 2018; ACS / Elsevier Outstanding Reviewer Awards; HIGHLY CITED Corresponding authors in The ROYAL SOCIETY OF CHEMISTRY (RSC), Cambridge, London; Top 10% cited article in the General Chemistry Section RSC Journal, Cambridge, London, Gandhian Young Technology Innovation Award (GYTI) 2020 and 2023, Selected Member of National Academy of Sciences (MNASc) etc. Dr. Chandra is also listed among the world's top 2% scientists from the last 5 years by Stanford University, USA.
